# A New Design of a CMOS Vertical Hall Sensor with a Low Offset

**DOI:** 10.3390/s22155734

**Published:** 2022-07-31

**Authors:** Fei Lyu, Shuo Huang, Chaoran Wu, Xingcheng Liang, Pengzhan Zhang, Yuxuan Wang, Hongbing Pan, Yu Wang

**Affiliations:** 1 School of Electronics and Information Engineering, Jinling Institute of Technology, Nanjing 211169, China; lyufei@jit.edu.cn (F.L.); huangshuo35@yeah.net (S.H.); chaoranwu@yeah.net (C.W.); xingchengliang1@yeah.net (X.L.); pzzhang@nju.edu.cn (P.Z.); 2 School of Electronic Science and Engineering, Nanjing University, Nanjing 210023, China; wangyuxuan@nju.edu.cn (Y.W.); phb@nju.edu.cn (H.P.); 3 School of Electronics Engineering, Nanjing Xiaozhuang University, Nanjing 211171, China

**Keywords:** vertical Hall sensors, seven-contact VHS, offset, current-related sensitivity, FEM simulations

## Abstract

Vertical Hall sensors (VHSs), compatible with complementary metal oxide semiconductor (CMOS) technology, are used to detect magnetic fields in the plane of the sensor. In previous studies, their performance was limited by a large offset. This paper reports on a novel CMOS seven-contact VHS (7CVHS), which is formed by adding two additional contacts to a traditional five-contact VHS (5CVHS) to alleviate the offset. The offset voltage and offset magnetic field of the 7CVHS are reduced by 90.20% and 88.31% of those of the 5CVHS, respectively, with a 16.16% current-related sensitivity loss. Moreover, the size and positions of the contacts are optimized in standard GLOBALFOUNDRIES 0.18 μm BCDlite^TM^ technology by scanning parameters using FEM simulations. The simulation data are analyzed in groups to study the influence of the size and contact positions on the current-related sensitivity, offset voltage, and offset magnetic field.

## 1. Introduction

Magnetic sensors have been widely used for applications in current sensing [1,2,3], biosensors [4], and consumer devices [5,6]. Hall effect sensors, which can be integrated into signal conditioning circuits using complementary metal oxide semiconductor (CMOS) technology, have the largest market share among all kinds of magnetic sensors [7]. Horizontal Hall sensors (HHSs), which are used to detect the magnetic field orthogonal to its surface, can meet the requirements of most application scenarios [8]. However, a three-dimensional (3D) Hall sensor is necessary in some applications, such as the measurement of a 3D magnetic field [9] and a space angle [10,11,12]. The most straightforward and promising way to implement 3D Hall sensors is to arrange multiple HHSs and vertical Hall sensors (VHSs) to detect magnetic fields orthogonal and parallel to the surface, respectively. VHSs with low sensitivity and especially large offsets are a weakness of the implementation of 3D Hall sensors [13,14,15].

After the development of the five-contact VHS (5CVHS) in [16], several variations have been proposed to improve the performance of VHSs, such as the four-contact VHS (4CVHS) [17,18], six-contact VHS (6CVHS) [19], and eight-contact circular vertical Hall devices (8CCVHD) [20]. The 5CVHS is a research hotspot to improve its performance [13,14,15], and variations have been proposed to reduce the offset by a large loss of sensitivity to accommodate low-voltage CMOS technology [21,22]. A symmetric four-folded and three-contact VHS (4F-3CVHS) has been proposed to reduce offset [23,24,25,26]. 4F-3CVHS, which has four active regions connected by additional wires, effectively improves VHS performance by increasing VHS complexity.

In this paper, we propose a new design for a seven-contact VHS (7CVHS) by adding two additional contacts in the body of a 5CVHS to reduce offset. The working principle of the 7CVHS and a contrastive analysis of the 5CVHS and 7CVHS are described in Section 2. The size and distances of contacts corresponding to the optimal performance of the 7CVHS are obtained by scanning parameters using FEM simulation, and the influence mechanism of the size and distances of contacts on the current-related sensitivity, offset voltage, and offset magnetic field are analyzed in detail in Section 3. Finally, Section 4 concludes this work.

## 2. From Five-Contact to Seven-Contact VHS

**Figure 1:**
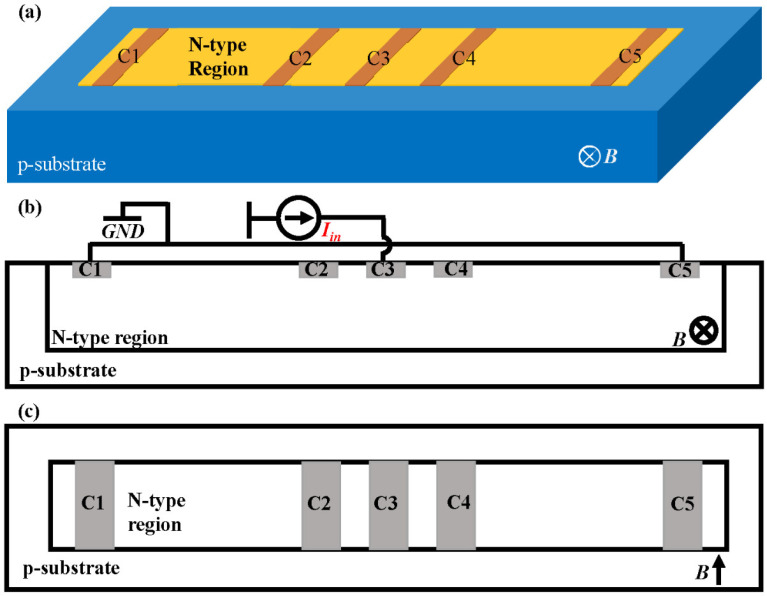
3D view (**a**), cross-section (**b**), and top view (**c**) of a 5CVHS implemented in CMOS technology.

A 5CVHS, as shown in Figure 1, contains an N-type region in the p-substrate and five n^+^-diffusions serving as contacts from C1 to C5. The N-doped well, which works as an active region, largely determines the performance of 5CVHS. When the 5CVHS is working, the outer contacts, C1 and C5, are short-circuited, and a biasing current is applied between C3 and C1 (C5), as shown in Figure 1b. Obviously, the voltages of contacts C2 and C4 are equal because of the symmetry of the structure of 5CVHS. When the 5CVHS is placed in magnetic field *B*, the voltages of contacts C2 and C4 change because of the Hall effect. The Hall voltage is measured between C2 and C4. The Hall voltages of C2 and C4 have opposite directions, so the Hall voltage of the 5CVHS can be expressed as:

(1) $$V_{H}(B\neq 0)=[V_{C2}(B\neq 0)-V_{C2}(B=0)]-[V_{C4}(B\neq 0)-V_{C4}(B=0)]$$

C2 and C4 serve as sensing contacts to obtain the Hall voltage of the 5CVHS. The voltage difference between them can be expressed as follows:

(2) $$V_{out}(B\neq 0)=V_{C2}(B\neq 0)-V_{C4}(B\neq 0)$$

Combining Equations (1) and (2), we can obtain the expression

(3) $$V_{out}(B\neq 0)=V_{H}(B\neq 0)+[V_{C2}(B=0)-V_{C4}(B=0)]$$

The main component of *V_out_* is the Hall voltage, from which the magnetic field can be calculated by their relationship, as follows:

(4) $$V_{H}(B\neq 0)=S_{I}I_{in}B$$

where *S_I_* is the current-related sensitivity of the 5CVHS, and *I_in_* is the bias current applied between C3 and C1 (C5). The other part of *V_out_* in Equation (3) is the voltage difference between C2 and C4 without a magnetic field, named the offset voltage *V_off_*. The voltages of C2 and C4 should be equal in an ideal situation because of the symmetry structure of the 5CVHS on both sides of C3. However, offset voltage is unavoidable in engineering practice. The offset voltage can be converted to an offset magnetic field *B_off_*, which is an important indicator of the sensor. *B_off_* can be calculated using the following expression:

(5) $$B_{off}=\frac{V_{off}}{S_{I}I_{in}}$$

**Figure 2:**
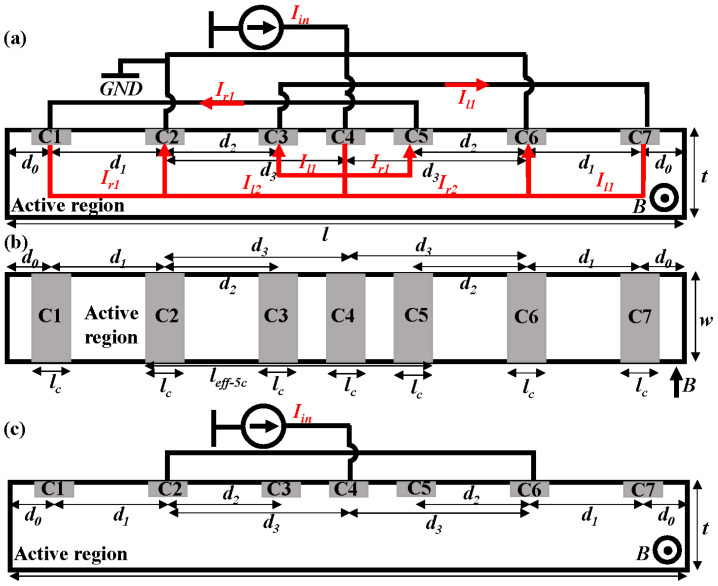
Cross-section (**a**) and top view (**b**) of the proposed 7CVHS. (**c**) 7CVHS in five-contact mode (5CVHS).

The offset is caused by asymmetry on both sides of C3 due to mask misalignment, the junction field effect (JFE), and piezoresistive effects. The new VHS, named the 7CVHS, shown in Figure 2a,b, introduces two connections on either side of C3 to neutralize the asymmetry and to lower the offset. The connections from C1 to C5 and C7 to C3, no doubt, lead to a decrease in the effective current of forming the Hall voltage. Thus, current-related sensitivity is reduced. The 7CVHS in the five-contact mode (7CVHS in 5C) in Figure 2c has the same characteristics as a 5CVHS. We compare a 7CVHS with the same size in the modes shown in Figure 2a,c to determine the advantage of the new VHS. Because the 7CVHS design is based on 5CVHS, it uses the same regions of technology. Additionally, 7CVHS is completely compatible with CMOS technology and contains an N-type region in the p-substrate and seven n^+^-diffusions serving as contacts from C1 to C7. The N-doped well works as an active region. An MVNVT is an N-type region, which is the most suitable area to implement the active region of the 7CVHS in standard GLOBALFOUNDRIES 0.18 μm BCDlite^TM^ technology [27]. The doping concentration and depth of the MVNVT are 1.413 × 10^17^ cm^−3^ and 1.018 μm, respectively.

**Figure 3:**
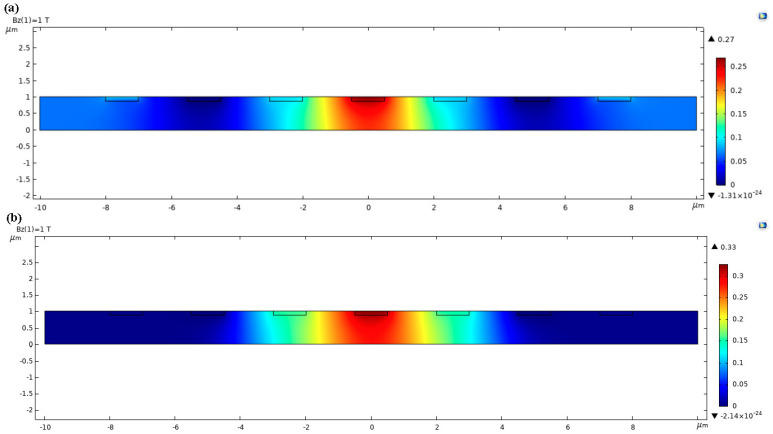
Models in COMSOL of 7CVHS (**a**) and 5CVHS (**b**), where the colors illustrate the voltage distributions.

In the COMSOL model of 7CVHS, MVNVT is used as the active region. The models in COMSOL of two 7CVHS types and the voltage distributions on their surfaces are shown in Figure 3. The simulation setup parameters are given in Table 1. The size and performance comparisons of the model in Figure 2 are listed in Table 2. The offset magnetic field, expressed in Equation (5), is affected by the current-related sensitivity *S_I_* and the offset voltage *V_off_*. The large current-related sensitivity, another important performance indicator of a VHS, is beneficial for suppressing the offset magnetic field. The biasing current and the magnetic field are set to 1 mA and 1 T, respectively, in the simulations to obtain current-related sensitivity. First, *d*_1_ changes to make C1 and C5 move away from C2 and C6 toward the left side of the 7CVHS when *l*, *d*_2_ and *d*_3_ remain the same to fix the positions of C2, C3, C4, C5, and C6. Second, *d*_2_ changes to make C3 move away from C2 toward C4 and C5 move away from C6 toward C4. The first step is repeated in one of *d*_2_. Third, *d*_3_ changes to make C2 and C6 move away from C4 toward both sides of the 7CVHS, and the second step is repeated on *d*_3_. Finally, *l* changes from 20 to 100 μm, and the third step is repeated on *l*. The offset is introduced by cutting C6 of 0.01 μm, and the size of the 7CVHS varies by *l*, *d*_1_, *d*_2_ and *d*_3_ operated by the above four steps to obtain the offset voltage in different sizes. We choose the structure of 7CVHS with the smallest value of *B_off_* and list it in Table 2. As illustrated in Table 2, the offset voltage and offset magnetic field of the 7CVHS are reduced by 90.20% and 88.31% of those of the 5CVHS, respectively, with a 16.16% current-related sensitivity loss. We also compare the 7CVHS with the design in [21]. As illustrated in Table 3, the offset voltage and offset magnetic field of the 7CVHS are reduced by 99.77% and 99.56% of those of the 5CVHS, respectively, with a 43.09% current-related sensitivity loss.

## 3. 7CVHS Optimization

The current flows are illustrated in Figure 2a. The bias current *I_in_* is divided into two currents to the left *I_l_* and to the right *I_r_* when passing contact C4. Then, *I_l_* has two current branches, *I_l_*_1_ from C4 to C3 and *I_l_*_2_ from C4 to C2. *I_l_*_2_ is the effective current for forming the Hall voltage of C3. *I_l_*_1_ introduced by the connection between C3 and C4 is beneficial to the suppression of the offset voltage. However, *I_l_*_1_, which has the same result as the short-circuit effect, reduces the effective current of the Hall voltage in C3. Thus, the introduction of *I_l_*_1_ reduces the Hall voltage of C3. *I_l_*_1_ flows from C3 to C7 through an external connection. Then, *I_l_*_1_ flows from C7 to the left and into C6. The current *I_r_* flowing to the right of C4 has the same characteristics. The Hall voltage measured between C1 (C5) and C7 (C3) is determined by the voltage variation caused by the Hall effect in contacts C1 to C7 and C3 to C5.

**Figure 4:**
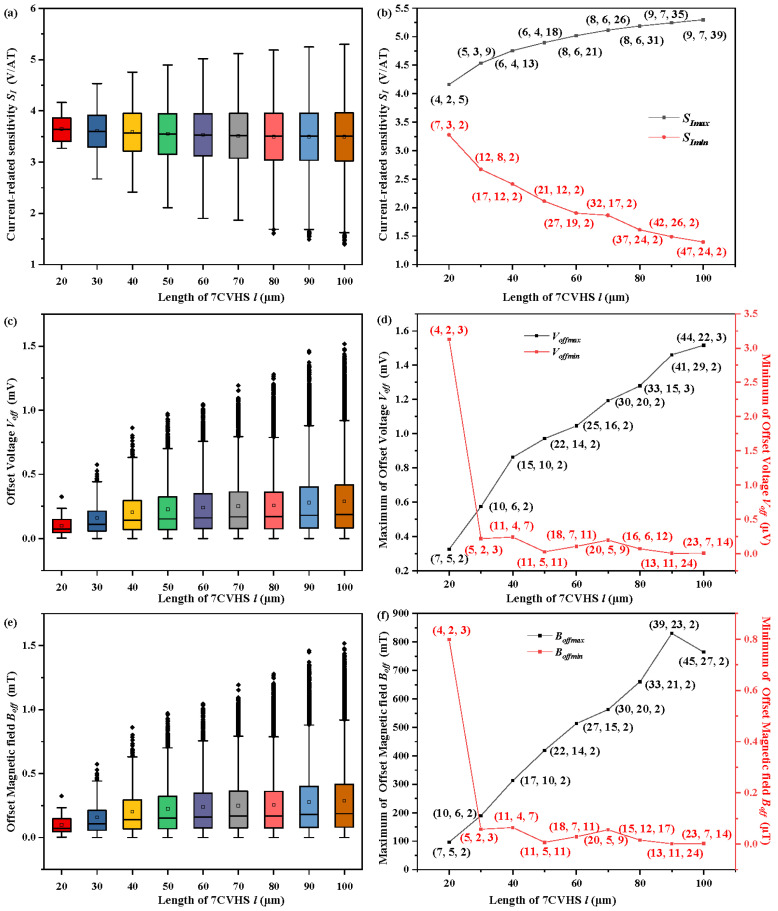
Current-related sensitivity *S_I_* (**a**), offset voltage *V_off_* (**c**), and offset magnetic field *B_off_* (**e**) of various lengths of the 7CVHS from 20 to 100 μm illustrated by boxplots. Maximum and minimum values of current-related sensitivity *S_Imax_* and *S_Imin_* (**b**), offset voltage *V_offmax_* and *V_offmin_* (**d**), and offset magnetic field *B_offmax_* and *B_offmin_* (**f**) in various lengths of the 7CVHS from 20 to 100 μm extracted from (**a**,**c**,**d**), respectively.

The current-related sensitivity *S_I_* of the 7CVHS of various lengths *l* are shown in Figure 4a. The maximum and minimum values of *S_I_* are extracted and shown in Figure 4b to clearly dictate the variation trend of *S_I_*. The other parameters *d*_3_, *d*_2_ and *d*_1_ corresponding to the maximum and minimum values of *S_I_* are marked by (*d*_3_, *d*_2_, *d*_1_) near the points in Figure 4b. The maximum value of *S_I_* increases due to its large length. The study and analysis in [27] confirm that the large length is beneficial to the increase in current-related sensitivity of the 5CVHS. The sensitivity of the 7CVHS, derived from the 5CVHS, is mainly determined by the Hall voltages of C3 and C5, which serve as sensing contacts in the 5CVHS. Thus, a large length contributes to the increase in the Hall voltages of C3 and C5. Although the large value of *d*_3_ makes the effective length of the 5CVHS in the 7CVHS containing C2, C3, C4, C5, and C6, the Hall voltages in C1 and C7 are weakened. However, the Hall voltage measured between C1 (C5) and C7 (C3) is determined by the voltage variation caused by the Hall effect in contacts C1 and C7, in addition to C3 and C5. Thus, the positions of C2 corresponding to the maximum values of *S_I_* are center-right between C4 and the left side of the 7CVHS to increase the Hall voltage in C1, and C7 and C5 are placed in the symmetrical position of C2 at the right side of C4. Moreover, the maximum values of *S_I_* correspond to the largest *d*_2_ to make C3 near C4. C3 has the largest Hall voltage when placed close to C4, as does C5. The last parameter *d*_1_ also should be the largest value to obtain the maximum values of *S_I_*. The large value of *d*_1_ makes C1 move away from C2 toward a lower *I_l_*_1_. However, the decrease in *I_l_*_1_ is beneficial to the increase in the Hall voltage in C5 and the measured Hall voltage. Moreover, *I_r_*_1_ and *I_l_*_1_ obtain large growth spaces with large lengths. The minimum values of *S_I_*, as shown in Figure 4b with a red line, decrease with increasing length *l* of the 7CVHS. The values of *d*_3_ corresponding to the minimum values of *S_I_* are the largest to make C2 move away from C4. Meanwhile, there are few spaces to place C1, and *d*_1_ has no other alternative but is set as 2 μm. Thus, the Hall voltages in C1 and C7 are suppressed to make the measurement low. Moreover, the values of *d*_2_, which determine the locations of C3 and C5, have a large influence on the Hall voltage in C3 and C5. The large *d*_2_ making C3 move toward C4 is beneficial to the original Hall voltage of C3 but causes a large *I_r_*_1_ and *I_l_*_1_ to lower the Hall voltage of C3. The values of *d*_2_ corresponding to the minimum values of *S_I_* make the C3 center-right balance the two above effects.

The offset voltages *V_off_* of the 7CVHS of various lengths *l* are shown in Figure 4c. The maximum and minimum values of *V_off_* are extracted and shown in Figure 4d to clearly reveal the variation trend of *V_off_*. Almost all values of *d*_1_ corresponding to the maximum *V_off_* are 2 μm to locate C1 near C2 and C7 near C6. The effective lengths of the 7CVHS from C1 to C7 are shortened in the above condition. A small effective length causes a large offset voltage [28]. The values of *d*_2_ and *d*_3_ are almost proportional to lowering the current *I_r_*_1_ and *I_l_*_1_ and to enlarging the offset voltage *V_off_* of the 7CVHS. The values of *d*_1_ corresponding to the minimum of *V_off_* are large to make C1 move away from C2 and C7 move away from C6 and increase the effective length of the 7CVHS, but they are not the largest because *I_r_*_1_ and *I_l_*_1_ decrease with increasing *d*_1_. Meanwhile, the values of *d*_2_ and *d*_3_ are set to make the current *I_r_*_1_ and *I_l_*_1_ as large as possible.

The offset magnetic field *B_off_* of the 7CVHS of various lengths *l* is shown in Figure 4e. The maximum and minimum values of *B_off_* are extracted and shown in Figure 4f to clearly reveal the variation trend of *B_off_*. *V_off_* and *B_off_* have almost the same relationship between the length of the 7CVHS *l,* and the values of *d*_1_, *d*_2_ and *d*_3_ corresponding to the maximum and minimum of *V_off_* and *B_off_* are almost the same. The offset voltage *V_off_* has a large effect on the offset magnetic field *B_off_* from the above comparison. The inflection point of the black line indicates that the current-related sensitivity *S_I_* of 7CVHS also affects the value of *B_off_*. The smallest value of *B_off_* is 0.8769 μT when the length of the 7CVHS is set to 90 μm.

**Figure 5:**
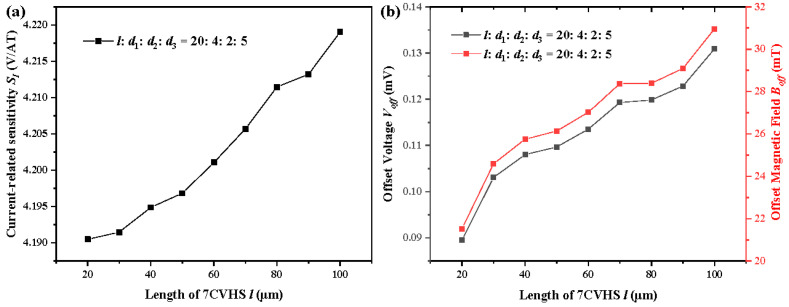
Current-related sensitivity *S_I_* (**a**), offset voltage *V_off_* and offset magnetic field *B_off_* (**b**) of various lengths of the 7CVHS from 20 to 100 μm. The ratio of values of *l*, *d*_1_, *d*_2_ and *d*_3_ is always maintained at 20:4:2:5.

The variations in *S_I_*, *V_off_* and *B_off_* in Figure 4 depend not only on the variational values of *l* but also on the changes in *d*_1_, *d*_2_ and *d*_3_. To concentrate on the impacts of the length *l* on *S_I_*, *V_off_* and *B_off_*, the length *l* changes from 20 μm to 100 μm with the same ratio of values of *l*, *d*_1_, *d*_2_ and *d*_3_ as 20:4:2:5. The values of *S_I_*, *V_off_* and *B_off_* with *l* from 20 to 100 and the same ratio of values of *l*, *d*_1_, *d*_2_ and *d*_3_ are illustrated in Figure 5. As shown in Figure 5a, the values of *S_I_* increase with a large length. The same ratio of values of *l*, *d*_1_, *d*_2_ and *d*_3_ keeps the current allocation unchanged. That is, the currents *I_r_*_1_, *I_r_*_2_, *I_l_*_1_ and *I_l_*_2_ largely remain the same. However, the large length causes the proportions of the contacts to decrease. This reduces the short-circuit effect. As shown in Figure 5b, the values of *V_off_* increase with a large length. A large length leads to a large resistance. In our design, a current source is used to bias the Hall sensor. Therefore, the supply voltage increases with a larger length, leading to a higher offset voltage. The gradient of *S_I_* is very small when compared with that of *V_off_*. The impacts of the length on the offset magnetic field are basically in line with those on the offset voltage, as shown in Figure 5b.

**Figure 6:**
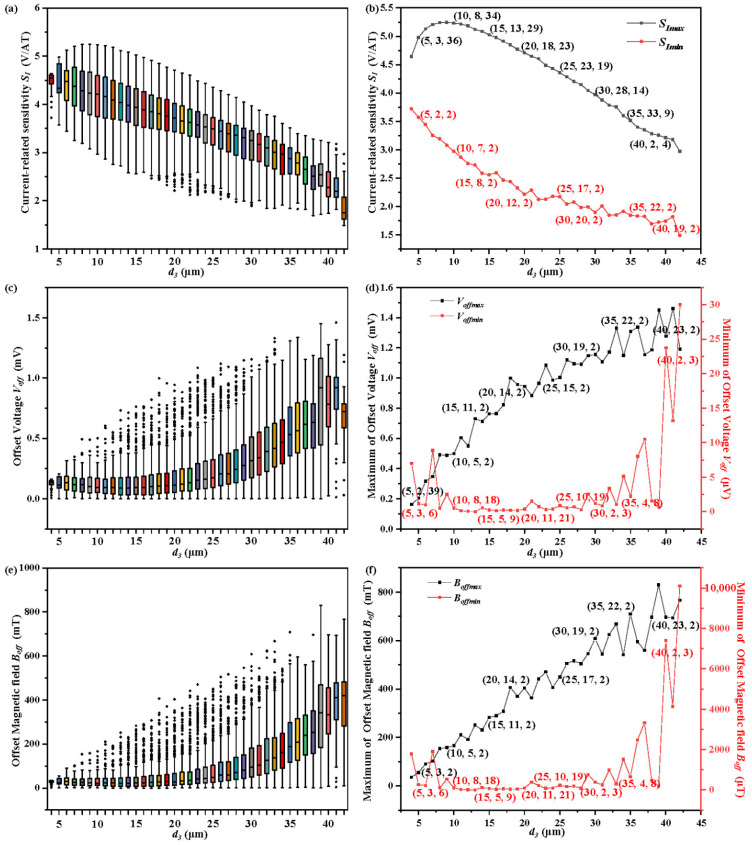
Current-related sensitivity *S_I_* (**a**), offset voltage *V_off_* (**c**), and offset magnetic field *B_off_* (**e**) of *d*_3_ from 4 to 42 μm with the length *l* set to 90 μm illustrated by boxplots. Maximum and minimum values of current-related sensitivity *S_Imax_* and *S_Imin_* (**b**), offset voltage *V_offmax_* and *V_offmin_* (**d**), and offset magnetic field *B_offmax_* and *B_offmin_* (**f**) in *d*_3_ from 4 to 42 μm extracted from (**a**,**c**,**d**), respectively.

When the length *l* of the 7CVHS is set to 90 μm, the current-related sensitivities *S_I_* of various *d*_3_ from 4 to 42 μm in different *d*_1_ and *d*_2_ are obtained, as shown in Figure 6a. The maximum and minimum of the current-related sensitivity *S_Imax_* and *S_Imin_* of each *d*_3_ are extracted and shown in Figure 6b to study the relationship between *S_I_* and *d*_3_. The values of *d*_1_ of *S_Imax_* are always large enough to lower the current *I_r_*_1_ and *I_l_*_1_ to obtain the largest *S_I_* in a fixed *d*_3_. When *d*_3_ is small enough, *l_eff-C5_*, the effective length of the 5CVHS in the 7CVHS, as shown in Figure 6b, is reduced, so the Hall voltages in contacts C3 and C5 are suppressed. *S_Imax_* achieves short growth with an increase in *d*_3_ by enlarging *l_eff-C5_*. However, *S_Imax_* decreases as *d*_3_ continues to increase. Meanwhile, the increases in *I_r_*_1_ and *I_l_*_1_ become the main factors that impact *S_Imax_* and make *S_Imax_* decrease with the increase in *d*_3_. Moreover, *S_Imin_* is obtained with the smallest value of *d*_1_ to lower the currents *I_r_*_2_ and *I_l_*_2_. The large *d*_3_ offers much space between C2 and C4 to place C3, and makes the current *I_r_*_2_ and *I_l_*_2_ decrease. The large *d*_3_ causes a small *S_Imax_* for this reason.

The offset voltage *V_off_* of the same length 90 μm with various *d*_3_ from 4 to 42 μm in different *d*_1_ and *d*_2_ are obtained, as shown in Figure 6c. The maximum and minimum values of offset voltage *V_offmax_* and *V_offmin_* of each *d*_3_ are extracted, as shown in Figure 6b, to study the relationship between *V_off_* and *d*_3_. The values of *d*_1_ are largely set as 2 μm to lower the currents *I_l_*_1_ and *I_r_*_1_ and bring about the largest offset voltage *V_offmax_*. *V_offmax_* shows a rising trend overall with increasing *d*_3_ because a large *d*_3_ enlarges the bias voltage of the sensor. A large bias voltage increases the voltage of all parts of the sensor, including the offset voltages. Similar to the analysis in Figure 4c, almost all values of *d*_1_ corresponding to the maximum of *V_off_* are 2 μm to move C1 near C2 and C7 near C6. The effective lengths of the 7CVHS from C1 to C7 are shortened in this condition to obtain a large offset voltage. The minimum values of the offset voltages appear with a large *d*_1_ to achieve a large effective length of 7CVHS.

The offset magnetic field *B_off_* of the 7CVHS of various lengths *d*_3_ is shown in Figure 6e. The maximum and minimum values of *B_off_* are extracted and shown in Figure 6f to clearly reveal the variation trend of *B_off_*. *V_off_* and *B_off_* have almost the same relationship between *d*_3_, and the values of *d*_1_ and *d*_2_ corresponding to the maximum and minimum values of *V_off_* and *B_off_* are almost the same. The offset voltage *V_off_* has a large effect on the offset magnetic field *B_off_* from the above comparison. This point confirms the analysis and results shown in Figure 4.

**Figure 7:**
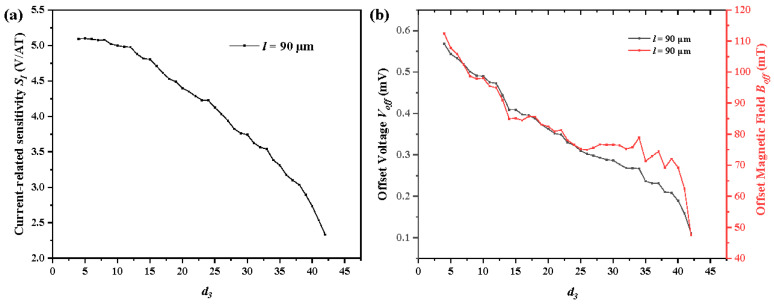
Current-related sensitivity *S_I_* (**a**), offset voltage *V_off_* and offset magnetic field *B_off_* (**b**) of *d*_3_ from 4 to 42 μm with length *l* set to 90 μm. The distances of C3 to C4, C4 to C5, C1 to the left boundary, and C7 to the right boundary are fixed.

To focus on the impacts of *d*_3_ on *S_I_*, *V_off_* and *B_off_*, we fix the distances of C3 to C4, C4 to C5, C1 to the left boundary, and C7 to the right boundary. In addition, the length of the 7CVHS is always set as 90 μm. Then, the value of *d*_3_ varies from 4 μm to 40.5 μm to simulate the values of *S_I_*, *V_off_* and *B_off_*. As shown in Figure 7a, the value of *S_I_* decreases with a large value of *d*_3_. With a large *d*_3_, the currents *I_r_*_2_ and *I_l_*_2_ decrease because of the increase in their corresponding resistances. Thus, *S_I_* decreases because of the reduction in the effective current of the Hall voltage. Moreover, the currents *I_r_*_1_ and *I_l_*_1_ increase because of the decrement of their corresponding resistances. Thus, the offset voltage decreases because of the large current needed to suppress the offset voltage, as shown in Figure 7b. The line of the offset magnetic field has a tendency similar to that of the offset voltage, as shown in Figure 7b. However, a small difference between them is derived from the variation in the value of *S_I_*.

**Figure 8:**
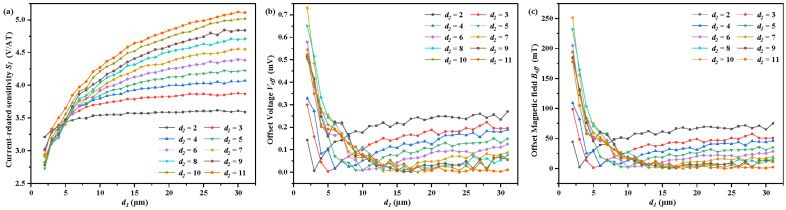
Current-related sensitivity *S_I_* (**a**), offset voltage *V_off_* (**b**), and offset magnetic field *B_off_* (**c**) of *d*_1_ from 2 to 31 μm with the length *l* and *d*_3_ set to 90 μm and 24 μm, respectively.

The relationship between the current-related sensitivity *S_I_* and *d*_1_ for various values of *d*_2_ is shown in Figure 8a. When *d*_2_ is unchanged, the growth of *d*_1_ is beneficial to the increase in *S_I_* by lowering the current *I_r_*_1_ and *I_l_*_1_. When *d*_1_ is small enough, the small *d*_2_ also leads to a large *S_I_* by lowering the current *I_r_*_1_ and *I_l_*_1_. When *d*_1_ becomes large, a large *d*_2_ brings about a large *S_I_* by making contacts C3 and C5 suitable to serve as sensing contacts with a large Hall voltage. The relationship between the offset voltage *V_off_* with *d*_1_ for various values of *d*_2_ is shown in Figure 8b. When *d*_2_ is small, *V_off_* decreases first with increasing *d*_1_ because a large *d*_1_ leads to low currents *I_l_*_1_ and *I_r_*_1_. Then, the large *d*_1_ brings about the large effective length of the 7CVHS to make *V_off_*. increase. When *d*_2_ is large, the increase in the effective length of the 7CVHS by enlarging the value of *d*_1_ always plays a leading role and determines the decrease in *V_off_*. The relationship between the offset magnetic field *B_off_* and *d*_1_ for various values of *d*_2_ is shown in Figure 8c. The values of *d*_1_ and *d*_2_ have the same effect on *V_off_* and *B_off_*, as seen by comparing Figure 8b,c. This point confirms the analysis and results shown in Figure 4 and Figure 6.

## 4. Conclusions

We have proposed a new design for a vertical Hall magnetic sensor with a low offset named 7CVHS. Two additional contacts are added to balance the asymmetry of the traditional 5CVHS. Comparing the 7CVHS with the 5CVHS shows that the offset voltage and the offset magnetic field are reduced by 90.20% and 88.31% of those of the 5CVHS, respectively, by a loss of 16.16% of the current-related sensitivity. Moreover, the length and distances of the contacts are optimized by scanning within the total coverage. When *l*, *d*_3_, *d*_2_, and *d*_1_ are set to 90, 13, 11, and 24 μm, respectively, the optimal performance of the 7CVHS is obtained, with the smallest offset magnetic field of 0.877 mT. In addition, the influence mechanisms of size and the distances of contacts on the current-related sensitivity, offset voltage, and offset magnetic field are studied by analyzing the data from different dimensions and perspectives.

## Figures and Tables

**Table 1:** Simulation setup parameters.

Symbol	Value	Description
*q* [C]	1.602 × 10^−19^	Electron charge
*n* [cm^−3^]	1.413 × 10^17^	Doping concentration
μ [cm^2^/Vsec]	1000	Electron mobility
*B* [T]	1	Magnetic field
*t* [m]	1.018 × 10^−6^	Depth of Hall sensor
*w* [m]	3 × 10^−6^	Width of Hall sensor
*I* [mA]	1	Bias current

**Table 2:** Size and performance comparison of the model in Figure 2.

Type of VHS	*l* (μm)	*d*_1_ (μm)	*d*_2_ (μm)	*d*_3_ (μm)	*S_I_* (V/AT)	*V_off_* (mV)	*B_off_* (mT)
**5CVHS**	90	13	11	24	5.94	4.45 × 10^−2^	7.50
**7CVHS**					4.98	4.36 × 10^−3^	0.877
					−16.16%	−90.20%	−88.31%

**Table 3:** Performance comparison with the design in [21].

Design	*S_I_* (V/AT)	*V_off_* (mV)	*B_off_* (*mT*)
**[21]**	8.75	1.93	197
**7CVHS**	4.98	4.36 × 10^−3^	0.877
	−43.09%	−99.77%	−99.56%
